# Design and rationale for the “Me & My Heart” (eMocial) study: A randomized evaluation of a new smartphone‐based support tool to increase therapy adherence of patients with acute coronary syndrome

**DOI:** 10.1002/clc.23254

**Published:** 2019-09-06

**Authors:** Florian Krackhardt, Lars S. Maier, Karl‐Friedrich Appel, Till Köhler, Alexander Ghanem, Carsten Tschoepe, Jürgen vom Dahl, Ralf Degenhardt, Anna Niklasson, Matti Ahlqvist, Matthias W. Waliszewski, Magnus Jörnten‐Karlsson

**Affiliations:** ^1^ Department of Internal Medicine and Cardiology Charité Campus Virchow‐Klinikum, Charité University Medicine Berlin Berlin Germany; ^2^ Klinik und Poliklinik für Innere Medizin II, Universitätsklinikum Regensburg Regensburg Germany; ^3^ Ambulantes Herzzentrum Kassel Kassel Germany; ^4^ Universitätslinikum Wuppertal Wupperthal Germany; ^5^ Asklepios Klinik St. Georg Hamburg Germany; ^6^ Kliniken Maria Hilf GmbH/Medizinische Klinik II Mönchengladbach Germany; ^7^ Institut für Klinische Forschung Herz‐ und Kreislaufzentrum GmbH Fulda Germany; ^8^ AstraZeneca R&D Gothenburg Gothenburg Sweden

**Keywords:** acute coronary syndrome, adherence, dual anti‐platelet therapy (DAPT), smartphone‐based support, study design, ticagrelor

## Abstract

A novel smartphone‐based patient support tool was developed to increase the adherence to antiplatelet therapy and lifestyle changes in patients after coronary angioplasty for acute coronary syndrome (ACS). The eMocial study (http://clinicaltrials.gov Identifier: NCT02615704) investigates whether an electronic support tool will improve adherence to comedication and lifestyle changes in ACS patients. The primary hypothesis of this trial is that an electronic support tool can increase adherence to comedication (primary endpoint) thereby supporting positive lifestyle changes (secondary endpoints). Patients hospitalized with ACS (ST elevation myocardial infarction [STEMI], non‐ST elevation myocardial infarction [NSTEMI], or unstable angina pectoris) and treated with ticagrelor coadministered with low‐dose acetylsalicylic acid will be randomized 1:1 to an active group receiving the patient support tool via a smartphone‐based application or to a control group without the patient support tool. Patient questionnaires to evaluate lifestyle changes and quality of life will be used at baseline and at the end of the 48‐week observation phase. Patients are asked to fill out questionnaires to determine their adherence, treatment attitudes, health‐care utilization and risk factors on a monthly basis. The study was started in February 2016 and the completion date is scheduled for October 2019. For final analysis 664 patients are expected be available. Preliminary baseline demographics were unstable angina pectoris (13.7%), NSTEMI (49.9%), STEMI (36.4%), male gender (86.3%), and diabetes mellitus (17.6%). Our study could significantly help to understand how inadequate adherence to antiplatelet therapy in ACS patients could be improved with a smartphone‐based application.

## 1 INTRODUCTION

In the United States, approximately 1 365 000 patients are hospitalized for acute coronary syndrome (ACS) annually.^1^ Despite optimal medical therapy, patients with recent ACS remain at high risk of recurrent coronary events.^2^ Therefore, long‐term management for patients discharged after ACS includes lifestyle changes (eg, physical activity plans, smoking cessation, and adherence to a healthy diet), the control of risk factors, and pharmacotherapy using antiplatelet therapy of acetylsalicylic acid (ASA), P2Y_12_ receptor inhibitors, beta‐blockers, statins, angiotensin‐converting enzyme inhibitors, or angiotensin receptor blockers.^3^

In clinical trials, therapy adherence tends to be high, whilst adherence in clinical practice is usually lower.^4^ A key strategy to enhance adherence is better communication and face‐to‐face coaching by a health‐care professional,^5^, ^6^ despite the fact that coaching sessions may not be sustainable from a cost perspective. Digital patient support has been proposed as a potential solution that could be effective at a sustainable cost.^7^, ^8^, ^9^

The purpose of the “Me & My Heart” (eMocial) study is to evaluate if patient support via a smartphone‐based app increases patients' adherence to treatment as compared to the current practice of providing information leaflets to the patients within the German health‐care system.

### 1.1 Rationale for the study design and focus on platelet inhibition

Current European Society of Cardiology (ESC) guidelines^3^, ^10^ recommend that antiplatelet therapy with low‐dose ASA, coadministered with P2Y_12_ receptor blockers, should be continued up to 12 months in ACS patients. However, real‐life comedication adherence seems to be an issue.^11^ In the PLATO trial,^12^ ticagrelor (90 mg twice daily) significantly reduced the rate of death from vascular causes, myocardial infarction and stroke without an increase in the rate of overall major bleeding. However, an increase in the rate of non‐procedure‐related bleeding as compared with clopidogrel in ACS patients was observed. The primary composite endpoint of cardiovascular death, myocardial infarction (MI), and stroke at 12 months showed a statistically significant benefit for ticagrelor to support the claim of a continuous benefit throughout the PLATO trial period. This suggests that the full benefit of ticagrelor treatment is achieved when it is continued for the full 12 months, or even longer based on the results of the PEGASUS trial. In the latter trial, ticagrelor (90 or 60 mg twice daily) was administered to patients between 1 and 3 years following MI.^13^ To achieve the full benefit of ticagrelor in clinical practice, treatment adherence and persistence should be at least similar as observed in clinical trials. The overall adherence or adherence rate to ticagrelor in the PLATO trial was 82.8%, and the median comedication duration was 277 days (interquartile range 179‐365).^12^ Limited data are available on ticagrelor adherence and discontinuation rates in general medical care. Moreover, reasons for discontinuation and clinical consequences from premature discontinuation have not been adequately studied.^14^ However, a 100% adherence to DAPT has been shown to significantly reduce the risk of death or secondary MI in stented patients^15^ indicating an unmet need to effectively enhance therapy adherence.

### 1.2 Patient adherence

Poor adherence to comedication or lifestyle changes are multifactorial and are the result of an interplay of patient‐, physician‐ and health‐care‐related factors.^16^ Among patients taking oral antiplatelet medication, a lack of awareness regarding their necessity is associated with patient‐reported non‐adherence.^9^ A recent systematic review on long‐term treatment of cardiovascular risk factors suggests cognitive education or behavioral counseling in multiple face‐to‐face meetings may improve that patients' adherence.^7^ Nevertheless, these approaches are not considered cost‐effective due to their resource‐intensive nature for patients and providers. Consequently, these practices have not been widely implemented into general health‐care practice.^7^

We sense that there is a need for alternative, cost‐effective methodologies to improve adherence, which can be customized to individual patient's needs and circumstances.^7^, ^17^, ^18^ A proposed strategy is the use of mobile health (mHealth) technology, which uses smartphone‐ or tablet‐based apps to reach diverse patient populations, including low‐income and otherwise difficult‐to‐reach patients.^19^ Significant improvements in treatment adherence using mHealth approaches have been observed in over 50% of randomized clinical trials for chronic diseases.^19^

### 1.3 Tools to assess therapy adherence

A broad range of direct and indirect methods is available to study adherence and persistence of a long‐term, oral drug treatment in clinical practice. However, there is currently no single, “gold standard” approach.^20^ Direct methods include laboratory testing of drug concentration or effect, such as inhibition of platelet activity (IPA). Indirect methods include the assessment of medication possession ratio (MPR), percentage of days covered (PDC), and patient‐reported outcome (PRO) instruments. PDC can be derived from the rates of prescription refills in pharmacy claims databases, or from medication event monitoring systems (MEMS) for pill counts via blister packs, bottles or electronic tablet dispensing devices.

Regarding our primary endpoint measure, there are several self‐reported adherence measures. The Morisky scale^21^ (MMAS) has been used for adherence evaluation in many studies and there are two versions of the MMAS, the four item (MMAS‐4) and eight item (MMAS‐8). It is a generic self‐reported scale^21^ which does not measure adherence per se, but rather medication taking behaviors like unintentional non‐adherence. According to today's standards, the MMAS is considered as non‐validated in terms of both content and from psychometric perspectives.^22^, ^23^ In studies measuring internal reliability for MMAS, many studies have shown low reliability and half acceptable reliability.^24^ However, no single method seems to stand out to deserve a “gold standard” designation.^11^ The PRO instrument used in this study was therefore based on a set of questions on adherence and has been designed for the current setting and measures adherence in a way that captures both intentional and unintentional non‐ adherence and was developed to make it specific for Dual Antiplatelet Therapy (DAPT) and to focus on true adherence irrespectively of the reason why patients did not take DAPT. In addition, we added standard questions regarding health‐care utilization and hospitalization which were part of the secondary endpoints.

The two major reasons for why we created our own instrument are 2‐fold. Today there are no existing PRO measures that are validated and measure adherence. Secondly, the goal of the study was to measure actual compliance which needed to be specific for antiplatelet therapy.

To our knowledge, no available instrument meets today's regulatory standards of being “fit for purpose.” Therefore, we developed a PRO instrument called the Brilique Adherence Questionnaire (BAQ). The BAQ (Appendix A1 and B1, Supporting Information) contains 15 questions measuring both intentional and unintentional non‐adherence. The first four questions refer to the number of tablets the patient has taken and the reasons for not taking them. Questions 5 to 11 capture intentional and unintentional non‐adherence using a three‐stage response option (“never/not at all” to “often/yes completely”).

### 1.4 Investigational app

A medical, smartphone‐based app, “Me & My Heart”, has been developed to increase treatment adherence (medication and lifestyle changes) by combining reminders about medication intake, information on the importance of treatment, motivation by supportive messages, and visualization of the effect of an individual's lifestyle choices on cardiovascular risk (specifically for patients with ACS who are prescribed drugs such as ticagrelor). The app is CE (Conformité Européene) marked as a class I medical device. The patient enters baseline information and the tool will then allow the patient to enter data on an ongoing basis and provide individualized feedback to the patient. The app will send daily optional reminders for medication intake, as well as motivational and informative messages every couple of days. The relationship between cardiovascular risks and lifestyle choices is graphically displayed in a qualitative way on a continuous basis.

In addition, the “Me & My Heart” app delivers monthly questionnaires to evaluate the trial endpoints. For the control group, the app will deliver only the questionnaires to evaluate endpoints and will not give any of the previously mentioned patient support. To assess if this information has an effect on the patients' lifestyle, we included an in‐house developed instrument, the Lifestyle Changes Questionnaire (LSQ; Appendix B1), which asks patients about diet, exercise and smoking habits. The 36‐item Short‐Form Health Survey was also included to understand the possible impact on health‐related quality of life (QOL).

## 2 METHODS

### 2.1 Study design and population

This “Me & My Heart” study (eMocial, http://clinicaltrials.gov Identifier: NCT02615704) is a randomized trial according to §23b Medizinproduktegesetz (German Medical Device Law). The primary objective is to evaluate the effect of patient support delivered through a smartphone‐based app on treatment adherence in ACS patients who received ticagrelor as their antiplatelet agent. More precisely, the primary hypothesis of this trial is that an electronic support tool can increase adherence to comedication (primary endpoint) thereby supporting positive lifestyle changes (secondary endpoints).

Treatment adherence will be measured using questions 1 to 4 in the BAQ every 4 weeks, and a scoring system for quantification from 0 to 14 will be employed. For the scoring system, one deduction for every missed ticagrelor tablet per week with a two‐tablet daily dosing, using a 7‐day recall period will be used and extrapolated to a 4‐week period.

Secondary objectives (Table 1) included the assessment whether the patient support delivered via the smartphone‐based app influenced the patient‐reported changes in disease understanding and treatment (assessed by BAQ questions 5‐11). Furthermore, the usefulness of this smartphone‐based app to improve drug adherence was part of the secondary objectives, that is, the time without antiplatelet medication according to BAQ/MEMS. Moreover, the change from baseline to the second visit in key risk factors was also studied. These risk factors included (a) blood pressure, and (b) laboratory parameters for low density lipoprotein (LDL) cholesterol, high density lipoprotein (HDL) cholesterol, glycated hemoglobin A1c (HbA_1c_), (c) body weight, waist circumference, body mass index (BMI), and (d) quality of life measures (assessed by SF‐36v2); lifestyle changes questionnaire (assessed by LSQ‐V1 and ‐V2); and (e) health‐care utilization (assessed by BAQ questions 12‐15). Details of the SF‐36v2 and LSQ‐V1 and ‐V2 scores are available in Table A1.

**Table 1:** Objectives

Primary objective:	Outcome measure:
To evaluate the effect of patient support delivered through an electronic device application on treatment adherence in patients with ACS prescribed ticagrelor in Germany.	Adherence to prescribed treatment according to questions 1‐4 in the BAQ, including a scoring system for quantification from 0‐14 (ie, one deduction for every missed ticagrelor tablet per week with twice‐daily dosing). The BAQ will be delivered via electronic device every 4 wk; the percentage of tablets taken during a 1‐wk recall period will be extrapolated to 4 wk.

Secondary objectives:	Outcome measures:
To evaluate the effect of patient support delivered via an electronic device application on adherence to ticagrelor medication.	Percentage of tablets taken during the 48‐wk observation phase measured by MEMS to record a time stamp for every tablet taken.
To evaluate the effect of patient support delivered via an electronic device application on the change from baseline to Visit 2 in key risk factors.	Blood pressure, laboratory parameters (if available: low‐density lipoprotein cholesterol, high‐density lipoprotein cholesterol, hemoglobin A_1c_, body weight and body mass index.
To evaluate the effect of patient support delivered via an electronic device application on quality of life.	Assessed by SF‐36v2®, at the start and end of the 48‐wk observation phase.
To evaluate the effect of patient support delivered via an electronic device application on patient‐reported lifestyle changes.	Assessed by LSQ‐V1 and ‐V2, at the start and end of the 48‐wk observation phase.
To evaluate the effect of patient support delivered via an electronic device application on patient‐reported disease understanding and treatment awareness.	Assessed by questions 5‐11 of the BAQ, delivered via electronic device every 4 wk.
To evaluate the effect of patient support delivered via an electronic device application on health‐care utilization.	Assessed by questions 12‐15 of the BAQ, delivered via electronic device every 4 wk.

Abbreviation: BAQ, Brilique Adherence Questionnaire; LSQ, Lifestyle Changes Questionnaire; MEMS, Medical Event Monitoring System; SF‐36, 36‐item Short‐Form Health Survey.

**Figure 1:**
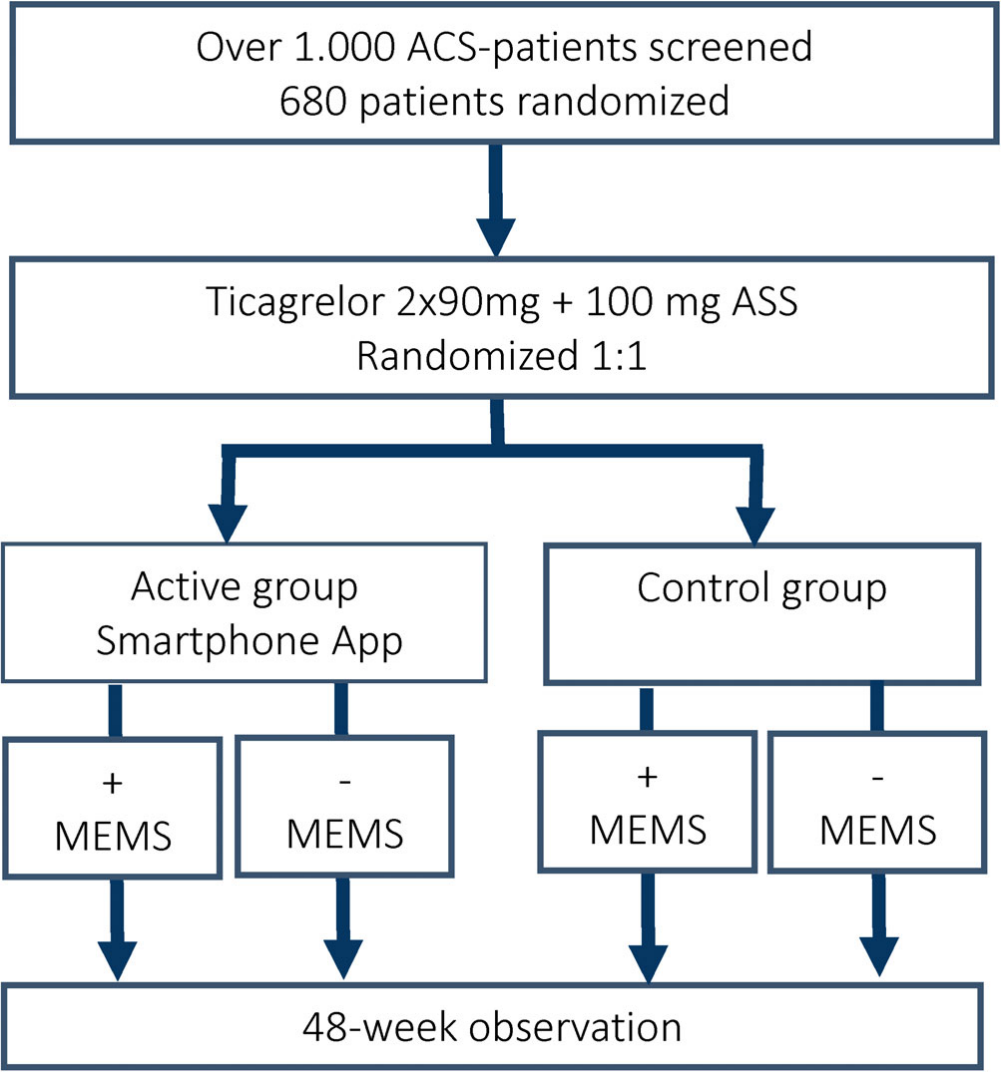
Study flow chart. ACS, acute coronary syndrome; BAQ, Brilique Adherence Questionnaire; GRACE, Global Registry of Acute Coronary Events; LSQ, Lifestyle Changes Questionnaire; MEMS, medication event monitoring system; NSTEMI, non‐ST segment elevation myocardial infarction; SF‐36, 36‐item Short‐Form Health Survey; STEMI, ST‐elevation myocardial infarction; UA, unstable angina pectoris

Before hospital discharge, patients diagnosed with ACS and treated with ticagrelor will be offered to participate in the study. It is planned that approximately 680 patients who give informed consent will be randomized 1:1 via an electronic case report form (eCRF) to a 2 × 2 factorial design with equal group sizes to receive the smartphone‐based patient support tool (with additional prompt questions for clinical evaluation) or a control smartphone‐based app used for data collection only (with no patient support). The detailed inclusion and exclusion criteria are described in Table 2. Both the active and control groups will be further randomized 1:1 into two subgroups. One subgroup will use MEMS to record when the patient takes their medication, and the other subgroup will not use the MEMS device (Figure 1). This study is being conducted in accordance with the current Declaration of Helsinki and is consistent with the International Conference on Harmonization and Good Clinical Practice guidelines.

**Table 2:** Inclusion and exclusion criteria

Inclusion criteria	Provision of patient‐informed consent prior to randomization. Female or male aged 18 years or older. Patients with ACS, diagnosed with STEMI, NSTEMI, or UA treated with ticagrelor before inclusion in the study and for whom the treating physician intends to continue prescribing twice‐daily ticagrelor coadministered with low‐dose acetylsalicylic acid, within 14 d following the diagnosis of the ACS event. Ability to read, understand, and write German. Patients must have access to a compatible electronic device and be willing to use it on a daily basis.
Exclusion criteria	Involvement in the planning and/or conduct of the study. Participation in another clinical study with an investigational product or medical device during the last 30 d, excluding prospective/retrospective register‐based studies that do not require any extra clinic visits in addition to ordinary healthcare. Patients being treated with oral antiplatelet drugs other than ticagrelor. Patients with contraindication to the use of ticagrelor. Patients with accepted/planned thoracic surgery (e.g. coronary artery bypass graft) or any other elective surgery that cannot be postponed until after study participation. Presence of serious/severe comorbidities in the opinion of the investigator which may limit life expectancy (< 1 y). Women who are currently pregnant or breast‐feeding.

Abbreviations: ACS, acute coronary syndrome; NSTEMI, non‐ST elevation myocardial infarction; STEMI, ST elevation myocardial infarction; UA, unstable angina pectoris.

### 2.2 Endpoints

The primary outcome variable is the percentage of tablets that patients report to have taken during the 48‐week observation phase, measured by the BAQ. The main secondary outcome variables include the percentage of tablets taken during the 48‐week observation phase, using the information from MEMS. Furthermore, the percentage changes from baseline to the second visit to determine key risk factors (including blood pressure and laboratory parameters), in quality of life (SF‐36v2, see Table A1) and in patient‐reported lifestyle changes (LSQ‐V1 and LSQ‐V2, see Table A1) were studied. Understanding of the disease and treatment awareness as assessed by the BAQ questions 5 to 11, health‐care utilization based on BAQ questions 12 to 15 and cardiovascular risk score (GRACE 2.0, see Table A1) complemented the secondary endpoints.^25^

### 2.3 Statistics and study analysis

The Student's *t* test assuming equal variances was used for the sample size calculations based on μ = percentage of tablets the patient reported to have taken. The test hypothesis was H_0_: μ_active_ = μ_control_ and H_A_: μ_active_ − μ_control_ ≥ 0.07 with a two‐sided α of 0.05 and a power of 85%. In other words, we test the hypothesis that the adherence rate is at least 7% higher in the smartphone‐supported patient group with a power of 85%.

Based on these assumptions, we calculated the 231 patients per arm. A 1‐year study of 243 randomized patients reported an approximately 5% drop out rate.^11^ However, a worst‐case dropout rate of 30% was assumed given the uncertainty of this novel approach, the setting of clinical practice within a observational framework, the potential technology barrier in the target population, and uncertainties of patient reporting in this study. The resulting final sample size was therefore estimated with 340 patients per arm with a total of 680 patients.

There are no substudies planned in the original protocol (http://clinicaltrials.gov Identifier: NCT02615704). However, post‐hoc analyses may be indicated depending on the findings of the planned study results within the framework of the a priori defined statistical analysis plan. Post‐hoc power calculations are planned for key secondary endpoints such as the percentage of tablets taken during the 48‐week observation phase and the percentage changes from baseline to the second visit in terms of key risk factors.

### 2.4 Study organization

This study was performed in compliance with Good Clinical Practice, including the archiving of essential documents. Data management, randomization, and statistical analysis were done by an independent third party. The study involved a steering committee consisting of five principal investigators as listed in Table B1.

## 3 PRELIMINARY RESULTS

Preliminary results are available in Table 3. As published at http://clinicaltrials.gov, the study was started in February 2016, whereas the actual primary completion date was reached in March 2019. The data validation and finalization as well as the analyses are ongoing until October 2019.

**Table 3:** Preliminary demographic data

Variable	All patients	Active group	Control group
Number of patients	664	332	332
ACS			
Unstable angina pectoris	91 (13.7%)	47 (14.2%)	44 (13.3%)
NSTEMI	331 (49.9%)	165 (49.7%	166 (50.0%)
STEMI	242 (36.4%)	120 (36.1%)	122 (36.7%)
Age (years) mean +/− SD	56.3 ± 9.5	56.6 ± 9.1	56.0 ± 9.9
Male gender	573 (86.3%)	284 (85.5%)	289 (87.0%)
Diabetes	117 (17.6%)	62 (18.7%)	55 (16.6%)
Hypertension	463 (69.7%)	235 (70.8%)	228 (68.7%)
Prior PCI	390 (58.7%)	199 (60.0%)	191 (57.5%)
Hyperlipidemia	380 (57.2%)	192 (57.8%)	188 (56.6%)
Obesity	178 (26.8%)	82 (24.7%)	96 (28.9%)
Medication			
Statin	564 (84.9%)	282 (84.9%)	282 (84.9%)
Beta‐blockers	528 (79.5%)	272 (81.9%)	256 (77.1%)
ACE inhibitor	435 (65.5%	219 (66.0%)	216 (65.1%)
Angiotensin receptor blockers	126 (20.0%)	66 (19.9%)	60 (18.1%)
Diuretics	89 (13.4%)	46 (13.9%)	43 (13.0%)
Calcium channel blocker	83 (12.5%)	43 (13.0%)	40 (12.0%)

## 4 DISCUSSION

Non‐adherence to comedication and lifestyle advice has a marked impact on therapy success and patients' outcomes. Adherence rates as low as 40% to 50% for long‐term treatment are typically reported in the literature whereas the impact is often underestimated in clinical practice and health‐cost estimates.^1^, ^26^, ^27^, ^28^, ^29^ There are various reasons for non‐adherence and not all reasons can be influenced by support tools or strategies. The primary objective of the eMocial trial is to evaluate the effect of patient support through a smartphone‐based app on treatment adherence. Our target group are patients with ACS who follow a DAPT regimen according to the most recent ESC guidelines.

### 4.1 Adherence tools

Consideration was given to different methods of tracking adherence when designing this study. A broad range of direct and indirect methods to characterize adherence are available but there are some challenges to accurately measure therapy adherence. The act of measuring patients' adherence is likely to have an impact on adherence activity. In addition, one can expect a potential motivational impact.

The more the measurement methodology influences patients' daily routines, the higher will be the adherence to treatment. It is therefore important to balance the requirement for highly granular, objective data with the need to interfere as little as possible with the results.

Objective and passive measures that collect data without the patient thinking about it would be the most desirable.

The MEMS device used in this study is also known as the “Helping Hand.” This device holds a medication blister, inserted by the patient, and registers the date and time whenever the medication is taken out from the package. Although originally designed as a tool to help improve adherence, it was adjusted so that it does not remind patients or visualize anything on the device. Data from a long‐term study have shown that the impact on adherence is only short term (less than 30 days),^30^ so the impact on a 12‐month study can be considered as small.

The use of PRO tools to capture adherence is common, but there are two major challenges with this approach. The first concern is data bias due to, for example, variable patient recall and social desirability (ie, patients feeling an expectation about how to answer questions). Because we are asking patients to report on something they are supposed to be actively doing (rather than something they experience or perceive), self‐reports on adherence tend to overestimate adherence. The second concern is the validity of current instruments. To our knowledge, there are no currently available adherence tools that would meet regulatory standards of being “fit for purpose” in terms of content and psychometric characteristics.^22^, ^23^ Thus, we designed our own instrument to measure both intentional and unintentional adherence for use in the eMocial study, the BAQ.

The advantage of using a smartphone‐based app (Appendix C) to judge therapy adherence in clinical practice is that actual clinic visits are not required. Furthermore, there are no clinical follow‐ups outside the standard of care during the 12‐month period and no follow‐ups if patients do not complete the surveys after initial enrolment in the study. Thus, the patients will only follow standard of care in the real‐life setting and improvements in adherence will be likely to stem from the benefits of using the smartphone‐based patient support tool app. Based on the same rationale, no prescribed treatment drug is provided in this study.

### 4.2 Limitations

One limitation of this study is the lack of closely comparable statistical data for study dropout and response rates to PRO tools delivered through the patients' own mobile devices. This means that the assumptions made for the sample size calculations are approximate. Consequently, there is a risk of not being able to establish statistical significance. In addition, because study‐specific health‐care professional interactions are minimized, there is a risk that patients in the study could stop responding to the PRO surveys even if they continue medication.

In terms of the postulated rather conservative dropout rate of 30%, as compared to the reference values discussed by Akl et al,^31^ we would like to propose a comparison of all patient demographics and risk factors in the patient group with and without follow‐up. If these comparisons do not reveal a higher risk profile for those patients who were lost to follow‐up, one could reasonably assume that difficult‐to‐treat patients are not excluded to present better outcomes.

In this study, the accuracy and granularity of adherence measurement are balanced with the risk of affecting the adherence by the act of measuring it. This is done by presenting BAQ every 4 weeks (ie, seldom enough to have a low effect on adherence) and then asking the patients for their number of missed doses in the past 7 days (ie, a short enough recall period for the patient to be able to remember). The weekly non‐adherence rate is then extrapolated to the full 4‐week period since the previous measurement. The fact that the PRO tools are new and not yet fully validated is also a limitation of this study. Because 50% of the study population use MEMS, cross‐correlation between the PRO and MEMS adherence numbers will be possible.

## 5 CONCLUSIONS

This study will evaluate how a mHealth solution can support ACS patients and positively impact their adherence to both medication and lifestyle changes over a clinically relevant treatment time of 12 months. If conclusive, our study could significantly help to understand how inadequate adherence to comedication could be improved with a smartphone‐based application.

## CONFLICT OF INTEREST

FK received lecture fees from AstraZenecax, MJ‐K, AN, MA are full‐time employees of AstraZeneca during the preparation of this manuscript. All other authors have no conflict of interest.

## APPENDIX A 1

**Table A1:** Other scores for secondary endpoints

SF‐36v2®	The SF‐36 consists of eight scaled scores, which are the weighted sums of the questions in their section. Each scale is directly transformed into a 0‐100 scale assuming that each question carries equal weight. The lower the score the higher is the degree of disability. The higher the score the less disability, that is, a score of zero is equivalent to maximum disability and a score of 100 corresponds to no disability. The eight sections are: vitality, physical functioning, bodily pain, general health perceptions, physical role functioning, emotional role functioning, social role functioning, and mental health.^32^
LSQ‐V1 and LSQ‐V2	Evaluation of lifestyle changes by Lifestyle Changes Questionnaire (LSQ)‐V1 and ‐V2 (patient‐reported outcome [PRO] instrument, developed for this trial). Questions with regard to a healthy diet, smoking behavior or regular exercise at the beginning (V1) and end (V2) of the trial to evaluate lifestyle modifications.
GRACE 2.0	GRACE is a risk score for ACS patients^25^ Inputs to calculate the mortality risk based on the GRACE score, which can range between 1 and 210. Inputs are age, heart rate, systolic blood pressure, amnestic presence of heart failure, prior MI, ST elevation, serum creatinine concentration, increased myocardial enzymes, and conducted coronary intervention

## APPENDIX B 1

**Table B1:** Study centers

No.	Study center	City	Principal investigator
01	Kliniken Maria Hilf	Mönchengladbach	Prof. Dr. Jürgen vom Dahl
02	Herzzentrum Wuppertal	Wuppertal	Dr. Till Köhler
03	Klinikum der Universität Regensburg	Regensburg	Prof. Dr. Lars S. Maier
04	Asklepios Klinik St. Georg	Hamburg	Dr. Alexander Ghanem
05	Zentralklinik Bad Berka	Bad Berka	Dr. Marc‐Alexander Ohlow
06	Herz u. Kreislaufzentrum Rotenburg a.d. Fulda	Rotenburg a.d. Fulda	Dr. Ralf Degenhardt
07	Ambulantes Herzzentrum Kassel	Kassel	Dr. Karl‐Friedrich Appel
08	Charité—Campus Virchow‐Klinikum	Berlin	PD Dr. Florian Krackhardt (coordinating investigator)
09	Klinikum Coburg	Coburg	Prof. Dr. Johannes Brachmann
10	Sana Kliniken Lübeck	Lübeck	Prof. Dr. Joachim Weil
11	Elbe‐Saale Klinik Barby	Barby	Dr. Henner Montanus
12	Charité—Campus Benjamin Franklin	Berlin	PD Dr. David Manuel Leistner
13	Universitätsklinikum Rostock	Rostock	Prof. Dr. Hüseyin Ince
14	Kliniken der Universität Heidelberg	Heidelberg	Dr. Markus Benhamin Heckmann
15	Klinikum Frankfurt‐Höchst	Frankfurt A.M.	Prof. Dr. Ulrich Hink
16	Universitätsklinikum SH, Campus Kiel	Kiel	Dr. Hans‐Joerg Hippe
17	Universitäts‐Herzzentrum—Bad Krozingen	Bad Krozingen	Prof. Dr. Dietmar Trenk
18	Klinikum Bernau—Herzzentrum Brandenburg	Bernau	Prof. Dr. Christian Butter
19	Praxis Dr. Woitge/Schiffer, Kleve	Kleve	Dr. Clemens Schiffer
20	Herz‐ und Gefäßzentrum Bad Bevensen	Bad Bevensen	Prof. Dr. Björn Remppis
21	Kardiologie—Gemeinschaftspraxis Kassel	Kassel	Dr. Frank‐Shephan Jäger
22	Klinikum Oldenburg	Oldenburg	Prof. Dr. Albrecht Elsässer
23	Klinikum Chemnitz gGmbH‐ und Chemnitz Küchwald	Chemnitz	Dr. Daniel Uhlemann
24	Herz‐ und Gefäßzentrum—Praxis Siegen	Siegen	Dr. Ulrich Overhoff
25	Medical Park Humboldtmühle Berlin	Berlin	Prof. Dr. Heinz Theres
26	Ev. Krankenhaus Düsseldorf	Düsseldorf	Prof. Dr. Ernst Vester
27	MediClin Fachklinik Rhein‐Ruhr Essen	Essen	Prof. Dr. Roger Marx
28	Universitätsklinikum Münster	Münster	Dr. Izabela Tuleta

Steering committee member	Name
Prof. Dr. Carsten Tschöpe	Charité Virchow Clinic, Berlin
Prof. Dr. Lars Maier	University Clinic Regensburg
Prof. Dr. Jürgen vom Dahl	Maria Hilf Clinics, Mönchengladbach
Prof. Dr. Heinz Theres	Rehab Clinic Humboldtmühle
Dr. Till Köhler	Helios Clinic Wuppertal

## APPENDIX C: APP STRUCTURE AND ITS DETAILS 1

- Education about disease and life style habits in five areas with a total of 22 chapters to describe medical treatment, disease information, and potential support to create new habits for example, exercise.
- Personalized reminders and motivational messages to drive suitable behavior and medication adherence. Personalization based on time since ACS, data in e‐diary and patients targets.
- DAPT medication management with reminders to take medication twice per day and an e‐diary to record when medication was taken. Reminders for prescription refill based the patients' records of medication adherence for 14 and 7 days before estimated stock depletion.
- e‐diary for patient biometrics such as blood pressure, blood lipids, and blood glucose (patient recorded) to support visualization of key parameters for the patient.
- e‐diary with option to set targets for exercise, body weight, and smoking. Personalized motivational messages were also used to promote life style changes (exercise level, body weight reduction, smoking cessation/reduction).
- A treatment index based on the patients' total activities to align with established treatment guideline (adhere to oral antiplatelet treatment, reduction in body weight if BMI > 25, smoking cessation and moderate exercise >150 minutes per week according to WHO recommendation). Individuals' development per week as well as comparison with the other patients' performance was given to recognize good work or stimulate further work if needed
- A screenshot for exercise summary is shown below:
